# FitTetra 2.0 – improved genotype calling for tetraploids with multiple population and parental data support

**DOI:** 10.1186/s12859-019-2703-y

**Published:** 2019-03-20

**Authors:** Konrad Zych, Gerrit Gort, Chris A. Maliepaard, Ritsert C. Jansen, Roeland E. Voorrips

**Affiliations:** 1 Groningen Bioinformatics Centre, University of Groningen, Groningen, The Netherlands; 2 Wageningen University and Research – Biometris, Wageningen, The Netherlands; 3 Wageningen University and Research - Plant Breeding, Wageningen, The Netherlands

**Keywords:** Genomics, Genotyping, Genotype calling, Polyploids, Autotetraploids, fitPoly

## Abstract

**Background:**

Genetic studies in tetraploids are lagging behind in comparison with studies of diploids as the complex genetics of tetraploids require much more elaborated computational methodologies. Recent advancements in development of molecular techniques and computational tools facilitate new methods for automated, high-throughput genotype calling in tetraploid species. We report on the upgrade of the widely-used fitTetra software aiming to improve its accuracy, which to date is hampered by technical artefacts in the data.

**Results:**

Our upgrade of the fitTetra package is designed for a more accurate modelling of complex collections of samples. The package fits a mixture model where some parameters of the model are estimated separately for each sub-collection. When a full-sib family is analyzed, we use parental genotypes to predict the expected segregation in terms of allele dosages in the offspring. More accurate modelling and use of parental data increases the accuracy of dosage calling. We tested the package on data obtained with an Affymetrix Axiom 60 k array and compared its performance with the original version and the recently published ClusterCall tool, showing that at least 20% more SNPs could be called with our updated.

**Conclusion:**

Our updated software package shows clearly improved performance in genotype calling accuracy. Estimation of mixing proportions of the underlying dosage distributions is separated for full-sib families (where mixture proportions can be estimated from the parental dosages and inheritance model) and unstructured populations (where they are based on the assumption of Hardy-Weinberg equilibrium). Additionally, as the distributions of signal ratios of the dosage classes can be assumed to be the same for all populations, including parental data for some subpopulations helps to improve fitting other populations as well. The R package fitTetra 2.0 is freely available under the GNU Public License as Additional file with this article.

**Electronic supplementary material:**

The online version of this article (10.1186/s12859-019-2703-y) contains supplementary material, which is available to authorized users.

## Background

Genetic studies in tetraploid species are lagging behind those of diploids. This is mostly due to the more complex genetics of tetraploids. Tetraploids have four copies of each of their chromosomes. Alleles at a marker locus can therefore be present in five different dosages: 0 (nulliplex), 1 (simplex), 2 (duplex), 3 (triplex) and 4 (quadruplex). Dosage scoring of such markers is challenging and has been computationally approached only since 2011 [1–4].

Automated dosage scoring is much needed in high-throughput genotyping technologies. In tetraploid species SNP arrays are the most widely used due to their cost efficiency [5]. SNP arrays like Illumina Infinium [6] or Affymetrix Axiom [7] measure the fluorescence signals of two dyes generated by the two alleles of each SNP. The higher the dosage of an allele, the stronger the fluorescence signal of the dye that is tracking it. Ideally the signal intensities should fall into one of the five possible categories. However, in practice these values are continuous and need to be converted into discrete dosage categories. Automated dosage scoring in tetraploid has been approached with k-means clustering [1] and hierarchical clustering [4] on data translated into polar coordinates, or Bayesian clustering [3].

The fitTetra software [2] uses another approach. First, the ratio of one of the signals to the sum of both signals is calculated. Ratio data is then arcsine-square root transformed to obtain approximately constant variance for the component distributions. Next, a mixture of normal distributions is fitted to the continuous ratio data, using the EM algorithm, and the fitted model is used to obtain categorical assignments. Similar approaches have been used in genomic research for many purposes, e.g. in scoring of AFLP markers [8–10]. This mixture model approach is computationally demanding but allows for automatic assignment of genotype categories by explicit modelling of the means of the distributions as a function of the allele ratios. The algorithm produces probabilities for each sample ratio to belong to the five distributions, and these can be used to assign a dosage to a specific class. This increases the reliability to deconvolute the distributions even if they overlap considerably. As a result, fitTetra is useful for automated dosage scoring in tetraploid species [11–14].

Data in multiple individuals for a single SNP can be modelled with a mixture of five normal distributions (each corresponding to a dosage score which may range from 0 to 4). The ratio values are from 0 to 1 (which corresponds to 0 to π/2 on the transformed scale) and the five distributions could be expected to be equally spaced along this range. However, in practice it is observed that they often are non-equally spaced. Moreover, limits of the ratios data range are often shifted towards the middle from one side or both [5]. These phenomena were accounted for in fitTetra [2] and further extended in an updated version of the software (fitTetra 1.0) [5]. That update added re-evaluation of the model if one of the extreme distributions is fitted with less than 2.5% of all samples and the adjacent distribution is fitted with more than 15% of samples. Such a situation is rarely expected. If the population under study is a full-sib family (FS), only one of the possible combinations of parental genotypes may result in a pattern close to such (Table 1 – AABB x AABB). Also for a collection of genotypes in Hardy-Weinberg equilibrium this is unlikely, as a low proportion of one of the extreme (nulli- or quadruplex) genotypes implies a low frequency of the corresponding allele and hence also a low proportion of the next (simplex or triplex) genotype. This re-evaluation strategy worked well for Illumina Infinium arrays [5]. Our recent analysis showed that even with this update, fitTetra1 may be misguided when shifts are severe, and that the update was insufficient to efficiently call genotypes for our Affymetrix Axiom array.

**Table 1:** Polysomic segregation ratios

Parent 1			Parent 2		
	0 = nulliplex	1 = simplex	2 = duplex	3 = triplex	4 = quadruplex
0 = nulliplex	1:0:0:0:0	1:1:0:0:0	1:4:1:0:0	0:1:1:0:0	0:0:1:0:0
1 = simplex	1:1:0:0:0	1:2:1:0:0	1:5:5:1:0	0:1:2:1:0	0:0:1:1:0
2 = duplex	1:4:1:0:0	1:5:5:1:0	1:8:18:8:1	0:1:5:5:1	0:0:1:4:1
3 = triplex	0:1:1:0:0	0:1:2:1:0	0:1:5:5:1	0:0:1:2:1	0:0:0:1:1
4 = quadruplex	0:0:1:0:0	0:0:1:1:0	0:0:1:4:1	0:0:0:1:1	0:0:0:0:1

Polysomic segregation ratios from random bivalent paring without double reduction for all possible combination of parental genotypes. For each parental combination the proportions of 0 (nulliplex): 1(simplex): 2 (duplex): 3 (triplex): 4(quadruplex) genotypes as expected in the offspring are shown

In order to improve the performance of fitTetra we added the possibility to use additional information. We allowed to specify subpopulations among the samples, which may be FS families (the direct F1 progeny of a cross between outbred, heterozygous parents, including the parents themselves) or panels of accessions such as collections of tetraploid breeding germplasm. Additionally, we made it possible to specify known genotypes for the parents of the FS populations.

The accuracy of dosage scoring with fitTetra increases with the number of samples analyzed. However, a combined analysis of samples from different types of populations is not straightforward. Full-sib families, used routinely in breeding of cross-pollinating polyploid crops, have expected allele ratios determined by the allele dosages of the parents. Collections of cultivars and breeding germplasm often display genotype frequencies close to those expected under Hardy-Weinberg equilibrium. However, assumptions concerning the combined allele dosage ratios can only be made if the composition of a dataset consisting of several FS families and/or cultivar panels is considered. To address this problem, we updated fitTetra to allow some model parameters to be shared between the subpopulations when they can be assumed to be equal (e.g. means and variances of the distributions which correspond to the allele dosage classes) while the mixing proportions of the dosages are specific for each subpopulation and therefore estimated separately. These additions result in increased power as combined analyses can be done comprising all samples, while using the most realistic assumptions for each of the subpopulations.

If the parental genotypes of a FS family are known, it is possible to calculate expected allele dosage ratios for their progeny, which can be used as starting values for the mixing proportions for that family in the model. Additionally, these known parental genotypes can be used to estimate starting values for the means of the distributions. This helps to guide the algorithm towards a better result. With our update to fitTetra (fitTetra 2.0, Additional file 1) that incorporates these capabilities, we were able to increase the call of SNPs by more than 25%, compared to version 1.0 (full data not shown).

Recently, another tool for automated genotype calling in tetraploids was published. ClusterCall [4] uses hierarchical clustering, with training and prediction phases. In the training phase, per probe, theta values (roughly equivalent to signal intensity ratios) from each of the FS families (offspring and parents) are clustered based on their intensity values, and dosages are assigned to the clusters based on expected FS segregation ratios. In the prediction phase, the theta values of the samples in the prediction set (e.g. a collection of breeding material) are clustered, and dosages are assigned by matching these clusters with those found in the training phase. A concordance metric, i.e. the proportion of training samples that were assigned the same dosage in training and test phase, is calculated and used for selection of reliable probes. The authors claim that ClusterCall is able to perform better than fitTetra 1.0 when multiple FS families are present. In our study we benchmarked ClusterCall against fitTetra 1.0 and fitTetra 2.0.

## Implementation

### Overview of fitTetra

The R package fitTetra [2] is a program for genotype calling of SNP markers in tetraploid species using normal mixture models. It consists of three main functions: 1) CodomMarker: the core function to fit a normal mixture model to a vector of transformed signal ratios of a single bi-allelic marker over all the samples; 2) fitTetra: a function to fit different normal mixture models to a single bi-allelic marker and select the optimal one; 3) saveMarkerModels: a function to fit mixture models for a series of markers and save the results to files. This suite of functions allows for automatic genotype calling for a collection of SNP markers. The normal mixture model is formulated for the response *y* = arcsine-square root transformed fraction s_b_/(s_a_ + s_b_) where s_a_ and s_b_ are the fluorescence signal strengths for alleles a and b. The normal mixture density of the *i*-th response *y*_*i*_ is $f\left({y}_i\right)={\sum}_{j=1}^5{\pi}_j{f}_j\left({y}_i\right)$ with *f*_*j*_(*y*_*i*_) the normal density with mean *μ*_*j*_ and common variance *σ*^2^. The probabilities *π*_*j*_, means *μ*_*j*_ and variance *σ*^2^ are estimated by maximum likelihood using the EM-algorithm. Starting values of the parameters can be provided by the user or are derived by the software through naïve clustering of the data. The genotype calling of observation *y*_*i*_ is based on the set of 5 values ${p}_{ij}={\pi}_j{f}_j\left({y}_i\right)/{\sum}_{j=1}^5{\pi}_j{f}_j\left({y}_i\right)$ (*j* = 1,..,5) that describe the probabilities that a sample belongs to each of the five distributions. If one of the *p*_*ij*_ is larger than a user-defined threshold value (e.g. 0.9) the genotype will be called to be *j-1* (as the distribution for *j = 1* corresponds to a dosage score of 0).

**Fig. 1:**
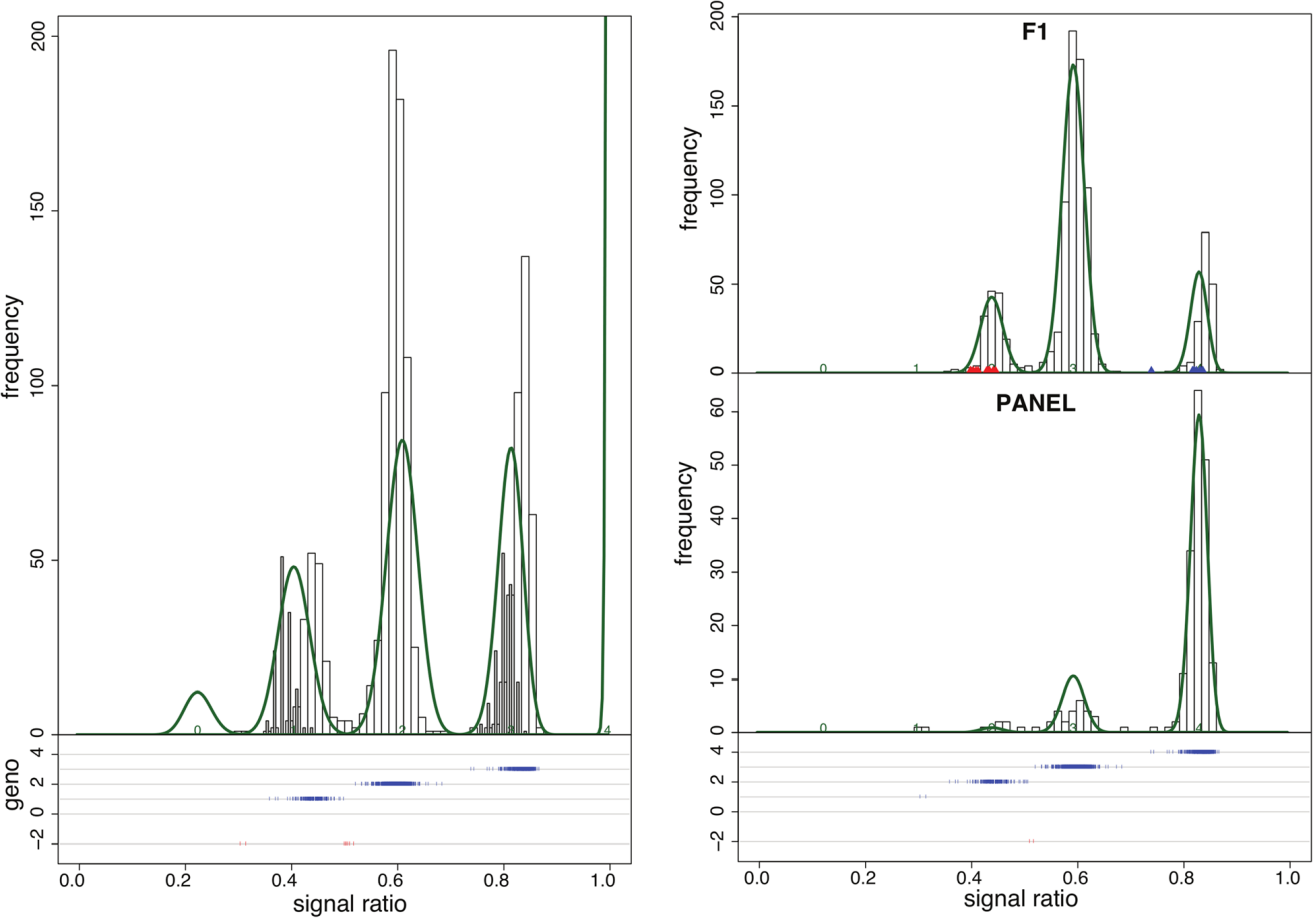
Histogram of genotype calling for probe_1028 (from fitTetra 2.0 test dataset), an example where the results of fitTetra 2.0 are different from fitTetra 1.0. Two populations are present: an association panel labeled “PANEL” and a FS family with two parental genotypes (both replicated). The association panel is assumed to be in Hardy-Weinberg equilibrium. *Right*: Calling with fitTetra 2.0 (using parental genotype data). The parental genotypes are set to be duplex (2) and quadruplex (4), leading to the segregation pattern 0:0:1:4:1, which is shown in the upper panel. *Left*: Calling with fitTetra 1.0. Without information on population structure, parental samples are genotyped as simplex (1) and triplex (3). Such a combination should result in a 0:1:2:1:0 pattern in the FS family. However, if we just consider samples from the FS family, the results of the calling suggest a 0:1:4:1:0 pattern which does not match any parental combination

The CodomMarker function allows restrictions to be placed on the *μ*_*j*_ and *π*_*j*_ parameters. Parameters *π*_*j*_ may be free or restricted to follow Hardy-Weinberg equilibrium. Parameters *μ*_*j*_ may be restricted such that 1) background signals for a and b (for the two SNP alleles) are equal or unequal; 2) the relationship between signal ratio and allele dosage is linear or quadratic. These restrictions allow the means *μ*_*j*_ to be asymmetrically positioned within the range 0 - π/2. An example of a histogram produced by fitTetra for a SNP for tetraploid potato is shown in Fig. 1. The dark bars in the histogram represent ratios of diploid potato genotypes included in this study. The allele ratios for diploid homozygotes are equal to ratios for homozygotes (nulliplex and quadruplex) in tetraploids and the ratio for the diploid heterozygote is the same as the ratio for duplex genotypes in tetraploids. Therefore, the position of diploid distributions compared to tetraploid distribution may serve as an extra check for the quality of the calling.

### Extension to multiple populations

In fitTetra 2.0 multiple populations may be specified, whereas fitTetra 1.0 treated all observations as stemming from one single population. When multiple populations are used, the model parameters for the means *μ*_*j*_ (i.e. the positions of the peaks) and variance *σ*^2^ (i.e. the width of the peaks on arcsine-square root scale) are shared among the different populations, while the mixing proportions *π*_*j*_ may be different for each population.

In fitTetra 1.0 the CodomMarker function, performing calling of a single marker allowed for three types of restrictions on the mixing proportions *π*_*j*_: 1) freely estimated (ptype = “p.free”), i.e. the only restriction is that ${\sum}_{j=1}^5{\pi}_j=1$; 2) Hardy-Weinberg equilibrium (“p.HW”) where only the allele frequency is estimated and mixing proportions are a function of those based on Hardy-Weinberg equilibrium, which is often reasonable for association panels; 3) fixed proportions (“p.fixed”), i.e. the *π*_*j*_ are kept fixed at their given values during the EM-algorithm (used when values are known from another source).

In the new version of the program we added a new type of restriction on the mixing proportions, p.type = “p.F1”, for an F1 population. Given the parental genotypes estimated in the previous iteration of the EM algorithm, the “p.F1” proportions are calculated as the expected tetrasomic segregation ratios of the F1 progeny of these two parents. These segregation ratios assume random bivalent pairing and no double reduction and are shown in Table 1. Double reduction is expected to occur in relatively low frequency even when there are occasionally multivalent pairings. The parental samples are specified as separate populations.

The EM-algorithm is used to find the maximum likelihood estimates of the parameters of the multi-population mixture model. In this algorithm E-steps (Expectation) and M-steps (Maximization) are repeatedly taken until the likelihood converges. In the E-step, given current estimates of the parameters, the probabilities for an observation to belong to each of the five mixture components are calculated. In the M-step, based on these calculated probabilities, new component means and variance are calculated over all observations. Then new mixing proportions are calculated per population depending on the type of restrictions on the mixing proportions, as discussed above.

In fitTetra 2.0, function “CodomMarker” (and, as a result the wrapper function “fitTetra” and the convenience function “saveMarkerModels”) produces the same type of output as fitTetra 1.0 with the exception of the output of probabilities of the five distributions, which are now population specific. Optionally, an array of histograms is plotted, showing per population (for association panels and for FS’s) the histogram of ratios with the fitted mixture model. Parental data are shown with separate coloured symbols in the histograms for the corresponding FS family (Fig. 1).

### Support for parental genotype data

When extra parental information is available, it can be used to further facilitate the genotype calling. Custom SNP arrays, the most popular means of genotyping tetraploids, are often based on discovery studies where parents of a population are sequenced in order to design the array for the population itself [5, 11]. The user can supply this information and set the probability type to “p.fixed” for the parental populations. This will fix the mixing proportions for the parental populations through the run of the algorithm. The probabilities (segregation ratios) of the corresponding F1 progeny will be calculated accordingly. It is possible that parental genotypes are erroneous (e.g. if they were derived from RNA-Seq they might be affected by differential gene expression). To avoid losing markers just because of that, fitTetra 2.0 will always attempt fitting a model where “p.free” is used instead of “p.fixed” and model with better BIC score will be selected. If parental genotypes supplied by the user are correct, the run with the “p.fixed” should produce model at least as good as with “p.free”.

Moreover, if the parents were analyzed in the same run as the FS family their dosages are also used to assign starting means for one or two of the mixing components. When parents have different dosages, two starting means are known, and the remaining three are estimated using a simple linear regression model. This may not be completely accurate as the relationship between dosage and intensity is not necessarily linear, but in our experience it provides a better start than the naïve clustering of the samples.

When multiple populations are measured in the same run, the means of the five genotype/dosage distributions are shared between populations. Because fitTetra 2.0 has the ability to process multiple populations at once, using known dosages of parents benefits the genotype calling not only for their F1 offspring but also for all the other populations, even when they are not related to these parents.

However, the starting parental dosages may be incorrect and applying these data as starting values may result in incorrect calls, or in not fitting any model at all. An example of this is shown in our previous SNP discovery study [5] in which we designed the array with RNA-Seq performance. When parental dosages are estimated from RNA-Seq data, they might be affected by differential expression of alleles (e.g. if the true parental genotype is AABB and allele A is expressed three times as much as allele B, the SNP array will report genotype AABB but the RNA-Seq read counts will suggest AAAB). In order to account for that, the algorithm fits a series of models both without and with parental dosage data; the best model (lowest value for Bayesian Information Criterion) is selected. For brevity, we will refer to fitTetra 2.0 without the use of parental data as fitTetra2NP and to fitTetra 2.0 where parental genotypes were used to fix the mixing proportions and calculate starting means as fitTetra2P later on.

## Results and discussion

We compared the performance of fitTetra 2.0 to fitTetra 1.0 and to the recently published ClusterCall [4] software based on two large datasets. One dataset was published as supplemental data for the ClusterCall manuscript. The other set is described in the section below.

### Test dataset

The test set (Additional file 2) comprised of 1000 randomly selected SNPs (with R base sample function [15]) from the STub, an Affymetrix Axiom 60 k SNP array. Parental genotypes were derived from the RNA-Seq SNP discovery study that led to the creation of the array. For the two parents, 12 and 13 replicate samples were used. The analysis of the test set is shown in the vignette accompanying the package. Analysis of the whole array with all the versions of fitTetra is not yet published.

In order to judge if genotype calling was performed correctly we made use of the fact that our collection comprises a FS family and its parents. For each SNP that was called, we compared the estimated genotype proportions in the offspring to the expected proportions from each possible combination of parental genotypes (using a chi-squared test for goodness-of-fit). If the parental combination that best matched the estimated proportions agreed with the parental genotypes that resulted from genotype calling we called the SNP “matching”. If this was not true, or not possible to assess (i.e. dosage not assigned to one or both of parents) the SNP was marked as “not matching”.

### Parameter selection

We compared the performance of fitTetra 2.0 to fitTetra 1.0 and to the ClusterCall [4] software. Both fitTetra and ClusterCall are sensitive to parameters used to perform the calling. For fitTetra, all default parameter settings were used, except for call.threshold = 0.75 and peak.threshold = 0.9.

In ClusterCall, the parameter min.posterior corresponds to the peak.threshold parameter of fitTetra and was initially set to the same value, 0.9. We tested number of values for min.posterior and max.range parameters. Based on the results we decided to use two values for min.posterior: 0.5 and 0.9 and one for max.range: 0.5. The code used for the parameter selection is attached in a form of an R script (Additional file 3) to assure easy replication of our results.

For the data from the ClusterCall paper [4] we followed the instructions in the package vignette. We again applied fitTetra with all default parameters, except for call.threshold = 0.75 and peak.threshold = 0.9. Since for these data no other source for the parental dosages was available, the corresponding capabilities of fitTetra2P could not be applied. The code used for the analysis is attached in a form of an R script (Additional file 4) to assure easy replication of our results.

### Comparison between fitTetra and ClusterCall

We benchmarked versions of fitTetra and ClusterCall by comparing total number of SNPs called and the number of called SNPs where genotypes of parents and offspring matched. Since we called parents together with the offspring we also checked the number of “matching” SNPs called.

When tested on the fitTetra2 test data, fitTetra2P was able to call more SNPs than other versions of fitTetra, and also more than ClusterCall with both parameter settings (Table 2). ClusterCall with min.posterior set to 0.5 was able to call more SNPs, but with a much lower proportion of “matching” SNPs. For this Axiom test data set all versions of fitTetra called more “matching” SNPs than ClusterCall.

**Table 2:** results of genotype calling on the test set of fitTetra 2.0

	fitTetra1	fitTetra2NP	fitTetra2P	ClusterCall min.posterior = 0.9	ClusterCall, min.posterior = 0.5
Called	63.4%	66.0%	69.0%	41.3%	77.0%
Matching	50.9%	57.4%	63.2%	20.5%	27.9%
Not matching	12.5%	8.8%	6.0%	20.8%	48.9%
Matching rate	80.3%	86.7%	91.3%	49.6%	36.3%
Not called	36.6%	34.0%	31.0%	58.7%	23.0%
> 90% in single category	13.7%	10.2%	12.3%	5.5%	1.9%
> 25% NAs	7.9%	20.9%	7.5%	–	–
No model	15.0%	2.7%	11.0%	53.2%	21.3%

ClusterCall performs training on separate FS families and uses the power of multiple FS’s to score dosage in unrelated collections [4]. In the fitTetra dataset however, there is only one large FS family and ClusterCall therefore cannot use concordance between families, and it performs worse than fitTetra.

Results of genotype calling on the test set of 1000 randomly selected probes included with fitTetra2. For ClusterCall the model is not fitted and when clustering is unsuccessful, the probe is returned with all scores missing so those were classified into “>25% samples unscorable” category.

When tested on the ClusterCall test data fitTetra2NP was able to call more SNPs and showed similar but higher accuracy to ClusterCall (Table 3). The data consists of three families. In all three fitTetra2NP was able to call more “matching” SNPs than ClusterCall, but in all the cases ClusterCall showed a higher proportion of “matching” calls.

**Table 3:** results of genotype calling on the test set of ClusterCall

	fitTetra2NP			ClusterCall		
	Fam.AxS	Fam.RxP	Fam.WxL	Fam.AxS	Fam.RxP	Fam.WxL
Called	87.5%	69.9%	88.9%	66.9%	51.5%	68.1%
Matching	73.2%	54.6%	71.2%	60.1%	45.2%	59.7%
Not matching	14.3%	15.3%	17.6%	6.8%	6.3%	8.5%
Not called	12.5%	30.1%	11.1%	33.1%	48.5%	31.9%
> 90% in single category	10.3%	27.9%	9.0%	6.1%	21.5%	4.8%
> 25% NAs	0.0%	0.0%	0.0%	0.0%	0.0%	0.0%
No model	2.2%	2.1%	2.1%	27.1%	27.1%	27.1%
Proportion of matching calls	83.7%	78.1%	80.2%	89.9%	87.8%	87.6%
Average proportion	80.6%			88.4%		
Average “matching”	66.3%			55.0%		

Results of genotype calling on the test dataset of ClusterCall. The calling was assessed for each of the three FS families separately. A x S – Atlantic x Superior, R x P – Rio Grande Russet x Premier Russet, W x L – Wauseon x Lenape.

Apart from the numbers of markers genotyped it is important to know the correctness of the genotyping. This is not straightforward as the true allele dosages are not known. However the overall correctness has an effect on downstream applications such as linkage mapping or GWAS. In Additional file 5 we present a comparison of GWAS results obtained with the fitTetra 2 test data as processed by ClusterCall and fitTetra 2.0. These results suggest that at least for some markers near the main QTL, the fitTetra scores are more accurate than those of ClusterCall.

## Discussion

### Improvement over the previous version

We updated fitTetra to support multiple populations, to model expected FS segregation ratios and to enable the use of parental dosage information to guide the calling. We tested fitTetra 2.0 against fitTetra 1.0, and showed a significant improvement in the performance. Since the previous version of fitTetra was used in a number of studies and proved useful and applicable in multiple data sets [2, 5, 13], this improvement is likely to benefit many researchers performing genetic analyses of polyploid species.

### Comparison with ClusterCall

We compared fitTetra 1.0 and fitTetra 2.0 with ClusterCall. In all the instances ClusterCall was able to perform the analysis much faster while fitTetra 2.0 was able to call the most SNPs with matching parental and progeny dosages. When tested on the ClusterCall test data, fitTetra 2.0 and ClusterCall showed similar performance. On the fitTetra 2.0 test data however, fitTetra 2.0 outperformed ClusterCall by far. This is not surprising as the main advantage of ClusterCall is the use of concordance between multiple FS. Therefore, where multiple FS are present ClusterCall is able to deliver accurate results swiftly. The mixture model based algorithm of fitTetra is computationally costly but able to perform reliably in more types of datasets.

### Using the match between the genotypes of parents and offspring for quality control

We showed that checking if genotypes of parents and offspring match is a valuable quality check. Non-matching parental and offspring dosages are an important warning. They can signify an erroneous calling procedure (e.g. convergence to sub-optimal results), technical artefacts in the data but also potential sample mix-ups/mis-labelling.

A limitation of the use of this approach is that some segregation patterns may be impossible to distinguish in smaller datasets (e.g. 1:8:18:8:1 and 0:1:2:1:0), so an apparent (non-)match between parents and offspring may not be completely certain.

### Extensions of fitTetra 2.0

The version of fitTetra presented here is only able to perform genotype calling in autotetraploids. An extension to any higher level of auto-polyploidy has in the meantime been implemented in a more advanced version of the package called fitPoly, available from CRAN (https://cran.r-project.org/package=fitPoly). The extension to allo-polyploids is less straightforward since some parental dosage combinations match with multiple segregation ratios.

Another possible extension would be the accommodation of (discrete) read count data from Next-Generation Sequencing, as opposed to (continuous) signal intensities from SNP arrays. This would involve a different model for the distribution of the data. However, recently several publications have already addressed this problem [16–18]. In [18] a comparison between the updog package and fitPoly was made. Not surprisingly fitPoly performed worse, as it was not designed to handle this type of data.

## Conclusions

The package fitTetra 2.0 provides the most robust approach for automated genotype calling in complex collections of autotetraploid samples. The tool is able to call large portion of SNPs correctly, even with strong confounding effects e.g. background signal, differences in performance between dyes or non-linear relationship between dosage and signal strength. Our tool is the most versatile and accurate solution for automated genotype calling in tetraploids.

## Availability and requirements

• Project name: fitTetra

• Operating system(s): Any platform for which the R [15] software is implemented, including Microsoft Windows and Linux. The software is included as an R package in Additional file 1.

• Programming language: R [15].

• Other requirements: None.

• License: GNU General Public License, version 2.

• Any restrictions to use by non-academics: None.

## Dataset

The test set comprises 1000 SNPs randomly selected from a custom Affymetrix Axiom 60 k SNP array. The population that was genotyped consisted of 1502 samples – 975 samples of the tetraploid FS population, 278 samples of the diploid FS population, parents of the tetraploid FS population in 12 and 13 replicates, parents of the diploid FS population in 3 and 2 replicates and 222 samples from a panel of breeding clones and market cultivars. The data set is available as Additional file 2.

## Additional files

Additional file 1: A tar archive containing fitTetra 2.0 package. (GZ 46494 kb)

Additional file 2: A zip archive containing all the data and code needed to run the comparisons. (ZIP 33420 kb)

Additional file 3: An R script containing all the code needed to run comparison between ClusterCall and fitTetra 2.0 on the dataset attached to fitTetra 2.0. (R 3 kb)

Additional file 4: An R script containing all the code needed to run comparison between ClusterCall and fitTetra 2.0 on the dataset attached to the ClusterCall manuscript. (R 6 kb)

Additional file 5: A small report of a GWAS analyses to compare the correctness of dosage calls by ClusterCall and fitTetra 2.0. (DOCX 531 kb)
