# Supplementary material for: FitTetra 2.0 – improved genotype calling for tetraploids with multiple population and parental data support
Source: BMC Bioinformatics. 2019 Mar 20;20:148. doi: 10.1186/s12859-019-2703-y (PMC6425654; doi:10.1186/s12859-019-2703-y)
Supplement: Supplementary file 5 — A small report of a GWAS analyses to compare the correctness of dosage calls by ClusterCall and fitTetra 2.0. (DOCX 531 kb) [file 12859_2019_2703_MOESM5_ESM.docx]

Supplementary file 5. The use of GWAS results as an indication of genotyping quality.

A genome-wide association study (GWAS) was performed, using genotypes produced for the test set of SNP markers by the fitTetra 2.0 and ClusterCall packages. We have used yield as a phenotype to test. The yield was estimated by weighting tubers produced by a plot of plants and converting to tons per hectare. GWAS was performed using R package GWASPoly [1] with all the standard settings. The results of the analysis are shown in Supplementary Figure 1.


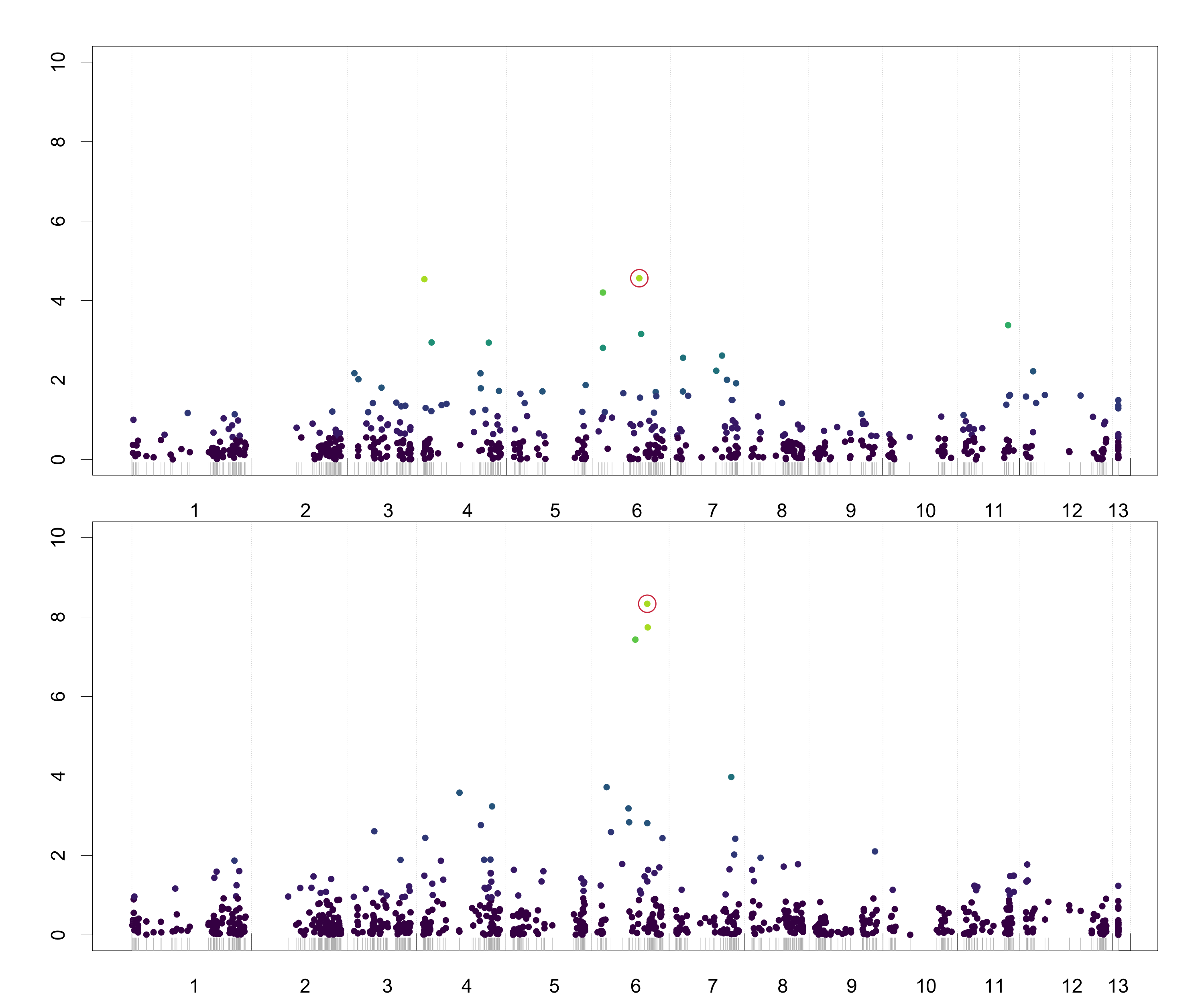


Supplementary Figure 1 – GWAS results for yield based on genotypes called by ClusterCall (top) and fitTetra 2.0 (bottom). X-axis – cumulative position in the genome, Y-axis - $-{log}_{10}(P)$. Each dot represents a marker, circle shows the marker with highest value of $-{log}_{10}(P)$.

As discussed in the main text, fitPoly2 was able to genotype more markers than ClusterCall. Also, the more significant P values obtained with some markers in the peak region on chromosome 6 suggest that the genotype calling of fitTetra 2.0 was more accurate.

1. Rosyara UR, De Jong WS, Douches DS, Endelman JB. Software for Genome-Wide Association Studies in Autopolyploids and Its Application to Potato. Plant Genome. 2016;9.
